# Facing CAR T Cell Challenges on the Deadliest Paediatric Brain Tumours

**DOI:** 10.3390/cells10112940

**Published:** 2021-10-29

**Authors:** Cristina Ferreras, Lucía Fernández, Laura Clares-Villa, Marta Ibáñez-Navarro, Carla Martín-Cortázar, Isabel Esteban-Rodríguez, Javier Saceda, Antonio Pérez-Martínez

**Affiliations:** 1 Translational Research in Paediatric Oncology, Haematopoietic Transplantation and Cell Therapy, Hospital La Paz Institute for Health Research, IdiPAZ, University Hospital La Paz, 28046 Madrid, Spain; cristina.ferreras@salud.madrid.org (C.F.); laura.clares.villa@idipaz.es (L.C.-V.); mcortazar.carla@gmail.com (C.M.-C.); 2 Haematological Malignancies H12O, Clinical Research Department, Spanish National Cancer Research Centre (CNIO), 28029 Madrid, Spain; lvfernandez@cnio.es (L.F.); mibanez@cnio.es (M.I.-N.); 3 Pathology Anatomy Service, University Hospital La Paz, 28046 Madrid, Spain; misabel.esteban@salud.madrid.org; 4 Department of Paediatric Neurosurgery, University Hospital La Paz, 28046 Madrid, Spain; javiermanuel.saceda@salud.madrid.org; 5 Paediatric Haemato-Oncology Department, University Hospital La Paz, 28046 Madrid, Spain; 6 Faculty of Medicine Universidad Autónoma de Madrid, 28029 Madrid, Spain

**Keywords:** paediatric, CNS tumours, CAR T cells

## Abstract

Central nervous system (CNS) tumours comprise 25% of the paediatric cancer diagnoses and are the leading cause of cancer-related death in children. Current treatments for paediatric CNS tumours are far from optimal and fail for those that relapsed or are refractory to treatment. Besides, long-term sequelae in the developing brain make it mandatory to find new innovative approaches. Chimeric antigen receptor T cell (CAR T) therapy has increased survival in patients with B-cell malignancies, but the intrinsic biological characteristics of CNS tumours hamper their success. The location, heterogeneous antigen expression, limited infiltration of T cells into the tumour, the selective trafficking provided by the blood–brain barrier, and the immunosuppressive tumour microenvironment have emerged as the main hurdles that need to be overcome for the success of CAR T cell therapy. In this review, we will focus mainly on the characteristics of the deadliest high-grade CNS paediatric tumours (medulloblastoma, ependymoma, and high-grade gliomas) and the potential of CAR T cell therapy to increase survival and patients’ quality of life.

## 1. Introduction

Central nervous system (CNS) tumours are the most common solid cancers in childhood, as well as the leading cause of cancer-related death in children. Paediatric CNS tumours comprise 25% of the childhood cancer diagnoses [1]. Current treatments are far from optimal. Despite their great heterogeneity, affecting their location and molecular mutations, all tumours are managed the same way: surgery, chemotherapy, and/or radiotherapy.

During the last decades, significant treatment advances have extended overall survival rates (60–70%), but outcomes for children with unresectable, relapsed, or refractory tumours remain dismal. Children with disseminated disease and younger age at the time of diagnosis have a particularly poor prognosis, with a 5-year survival of 15–30% [2,3]. Even the cure is paid at a high price; the adverse effects of current treatments on the developing brain leave these children with long-term dramatic sequelae. Their quality of life is drastically affected by neurologic irreversible effects, endocrine disease, cognitive and developmental disorders, and the possibility of generating secondary malignancies [4].

Paediatric CNS tumours have not been as extensively studied as the adult ones. They differ in many aspects, such as clinical presentation, histological distribution, gene mutations, embryological origins, location, and the tumour microenvironment (TME), making them respond differently to the treatments [5,6]. Moreover, we must consider that the paediatric immune system is developing and age-dependent, leading to differences in treatment response [7].

Advances in molecular characterisation in the last years have brought the identification of new potential targeted molecular therapies that are being tested in early-phase clinical trials [5,8].

Despite this, the long-term survival of these young patients remains unacceptably low. Furthermore, the use of pharmacologic therapies for paediatric brain cancers can be particularly challenging due to the low permeability of the brain–blood barrier (BBB) and the lack of approved novel agents [9].

Recently, with the increased understanding of CNS immunology, along with the reported clinical success of chimeric antigen receptor T (CAR T) cells in haematological malignancies, CAR T cell immunotherapy has opened new therapeutic avenues for a targeted approach to eliminate cancer cells while sparing healthy brain tissue.

However, paediatric brain tumours represent a challenge for successful immunotherapy treatment. They possess unique characteristics, including low mutational burden, tumour heterogeneity that leads to tumour evasion, location, the barrier generated by the BBB, the immunosuppressive TME, and the treatment-related toxicities that may cause fatal consequences on the CNS [10].

In this review, we will focus mainly on the challenges of CAR T cell therapy in the more prevalent high-grade CNS paediatric brain tumours, such as medulloblastoma, ependymoma, and high-grade gliomas. Atypical teratoid/rhabdoid (AT/RT) is also one of the deadliest brain tumours but, also, it is very rare [11]. We are going to discuss the potential strategies to overcome these hurdles.

## 2. CAR T Cell Immunotherapy

Adoptive cell therapy (ACT) is one of the most promising strategies used in cancer immunotherapy [12]. ACT consists of the transfer of immune cells to a patient after selection, genetic manipulation, and ex vivo expansion to enhance antitumour activity. ACTs include tumour-infiltrating lymphocytes (TILs), T cell receptor (TCR) T cells, CAR T cells, and natural killer (NK) cells [13]. ACT has achieved high regression rates in several cancer types; however, lower rates have been reported in others, especially solid cancers [14,15]. Within ACT therapies using effector T cells, CAR T cells have emerged as a powerful strategy harnessing the power of the immune system to eradicate cancer [16].

CAR T cells involve the genetic modification of autologous or donor T cells to recognise a specific antigen. CARs incorporate an extracellular antigen-binding domain, usually derived from a single-chain variable fragment (scFv) of a monoclonal antibody, which is fused via a transmembrane linker to an intracytoplasmic signalling domain (CD3z). This basic engineered construct forms a first generation CAR. The addition of one or two costimulatory domains, which are needed to promote T cell activation and functionality, create second and third generation CARs, respectively [17,18]. Additionally, CAR T cells can be used as delivery vehicles of inflammatory cytokines (IL-7, IL-12, IL-15, IL-18, IL-23), antibody fragments, or other biomolecules that enhance their antitumour activity, known as fourth generation, armoured CAR T cells or TRUCKS-T cells redirected for universal cytokine-mediated killing [19,20]. Upon antigen recognition, the intracellular signals trigger CAR T cell activation, which, in turn, secrete perforin/granzyme and inflammatory cytokines, leading to tumour cell killing. Besides recognising tumour specific antigens, CAR T cells can also target tumour-associated antigens (TAAs) and immunosuppressive cells within the TME.

Nowadays, there are five CAR T cell therapies approved by the Food and Drug Administration for refractory large B-cell lymphoma, acute lymphoblastic leukaemia, mantle cell lymphoma, follicular lymphoma, and multiple myeloma. In this context, anti-CD19 CAR T cells have demonstrated consistent antitumour efficacy in children and adults affected by relapsed B-cell malignancies, with the percentage of complete remissions ranging from 70 to 94% in the different trials [21]. Even though there has been remarkable success in B-cell malignancies, clinical trials testing CAR T cells for solid tumours have only reported sporadic and transient objective responses [22,23,24,25,26,27,28]. CAR T cells offer some advantages over chemotherapeutic drugs, including targeting multiple tumour antigens [29]. Nevertheless, lack of specific tumour antigens, inefficient traffic of CAR T cells to the tumour site, or the hostile TME that avoids T cell activation, proliferation, and survival are some of the issues accounting for the failure of CAR T cell therapy in solid tumours. In the specific case of brain tumours, other aspects must be considered, such as the difficulty to trespass the BBB or the need to avoid inflammation or any other treatment-related toxicity. In this regard, Abramson et al. reported CD19 CAR T cells could target CNS diffuse B-cell large lymphoma cells, proving that CAR T cells can bypass the BBB and encouraging further research for using CAR T cells for brain tumours [30]. Others have also shown the efficient trafficking of CAR T cells to the paediatric CNS [31,32,33].

For paediatric patients, additional caution is required, as their brain is in development and any damage may cause life-long side effects [34].

Most of the clinical trials are generally focused on adult patients, mainly in glioblastoma (GBM), showing a modest efficacy [26]. However, these studies have served as a demonstration of the feasibility and safety of CAR T cells to treat brain tumours, paving the way to extend further the research in this field and to include more paediatric patients. As a result of this increased interest in testing CAR T cell therapies in paediatric CNS tumours, a search in clinicaltrials.gov typing “CAR” and “brain tumours” shows a total of 10 registered trials actively recruiting patients (Table 1).

## 3. Overview of High-Grade CNS Paediatric Tumours

In this section, we will give a rough summary of the most common high-grade CNS tumours in children. There is an extensive bibliography on this topic; since that is beyond the scope of this review, see [35,36,37] for an overview.

### 3.1. Medulloblastoma

Medulloblastoma (MB) is a highly aggressive embryonal neuroepithelial tumour (World Health Organization (WHO) grade IV) that usually arises from the cerebellum or dorsal brainstem. MB accounts for nearly 20% of all CNS tumours in children. Current standard therapies based on surgery, chemotherapy, and radiotherapy have increased the survival rate but with devastating long-term toxicities [38]. WHO classifies MB into four histological groups: large cell and anaplastic, nodular desmoplastic, extensive nodularity, and classic. Additionally, MB is divided into four molecular subgroups: wingless/integrated (WNT), sonic hedgehog (SHH), group 3, and group 4 [36,39]. MB presents great heterogeneity, even within each subgroup. These subgroups differ in many features, such as cells of origin, tumour vasculature/architecture, molecular mutations, alterations in epigenetic regulators, and prognosis [5,36,40].

The WNT subgroup is characterised by the activation of the WNT pathway. It comprises 10% of all MBs and has the best clinical outcome prediction, with a 5-year overall survival (OS) >95%.

The SHH subgroup comprises very heterogeneous tumours that share the overexpression of the SHH pathway. This subgroup represents 30% of all MB, with a 5-year OS of 70%.

Group 3 represents 25% of all MB and has the worst prognosis among the subgroups, with a 5-year OS of 40–50% and half of the patients presenting metastasis at diagnosis. The underlying molecular drivers have not yet been characterised, although MYC amplification and gain or loss of chromosome function confers a poor prognosis.

Group 4 is the least understood group but the most prevalent. This group represents 35% of all MBs, with a 5-year OS of 75–90% [8,40]. Multiple histone mutations and epigenetic aberrations have been found in this subtype.

Some targeted therapies in early clinical trials for the SHH group and decreases in the chemo and radio doses are being implemented to achieve the same efficacy as that achieved for the WNT group, but there are no specific treatments developed for groups 3 and 4 [5,36].

### 3.2. Paediatric High-Grade Gliomas

Paediatric high-grade gliomas (pHGGs) comprises four types: diffuse midline glioma, H3 K27-altered; diffuse hemispheric glioma, H3 G34-mutant; diffuse paediatric-type high-grade glioma, H3 wildtype and IDH wildtype; and infant-type hemispheric glioma [41], as classified by the WHO [39], and represent 8–12% of all primary brain tumours in children. Of note, glioblastoma is no longer used in the setting of a paediatric-type neoplasm. pHHGs are very aggressive, with a low survival rate. Despite numerous approaches and molecular data, the 5-year survival rate is still ranging 15–35% [41,42]. The treatment involves a combination of surgery, radiation, and chemotherapy. Histologically, pHGG are identical to the adult HGGs, but the molecular genetics and genomic alteration patterns are unique. Epigenetic changes are common in these tumours. pHGGs carry different and mutually exclusive histone gene mutations specific to tumour location, receptor tyrosine kinase mutations, and mutations in the tumour suppressor gene TP53. pHGGs present with extensive tumour heterogeneity. Although different pHGG subgroups have been proposed in terms of anatomical location, clinical outcome, histone mutations, or pathway alterations, their great heterogeneity complicates their classification and treatment [8,43,44,45,46]. In addition, the molecular genetics of pHGGs differ between infant and older children with HGG [47,48]. Targeted therapies are also under investigation in early clinical trials but, to date, they have not conferred a higher survival, even in combination with standard treatments [35,49].

Diffuse midline gliomas (DMG) are rare tumours characterised by their aggressiveness and their infiltrative growth pattern [50]. DMGs represent 80% of all paediatric brain tumours that occur in the brainstem. DMGs have no cure and only less than 10% of patients survive beyond 2 years from the time of diagnosis [51]. Their treatment remains very challenging since their location within the brainstem makes surgical resection inappropriate. Radiotherapy is the standard of care and the combination with chemotherapy has not shown any benefit. Around 85% of DMG carry a K27M mutation in histone H3 gene or, less commonly, in the related HIST1H3B gene [52].

### 3.3. Ependymomas

Ependymomas account for 10% of primary intracranial tumours in children, being the third most common CNS tumour in this age group. They locate in the brain, posterior fossa, spinal cord, and supratentorial region. There are nine subgroups based on molecular analysis depending on the age, location, and biology but, even within those subgroups, there are subtypes with dismal differences in prognosis. Two-thirds of the tumours are in the posterior fossa. Posterior fossa ependymoma group A (PFEPN-A) is the most aggressive but molecular genetic studies have shown a very low mutational burden, with low somatic mutations. Posterior fossa ependymoma group B (PFEPN-B) tumours display frequent large-scale copy number gains and losses, and have better outcomes. More than 70% of supratentorial ependymomas are defined by highly recurrent gene fusions in the NF-κB subunit gene *RELA* (ST-EPN-RELA) [36,53,54]. The current standard of care is tumour resection and radiation, with chemotherapy giving no substantial survival advantages.

## 4. Challenges and Opportunities of Car T Cell Therapy in Paediatric CNS Tumours

### 4.1. Challenges for Paediatric Brain Tumours

**Figure 1:**
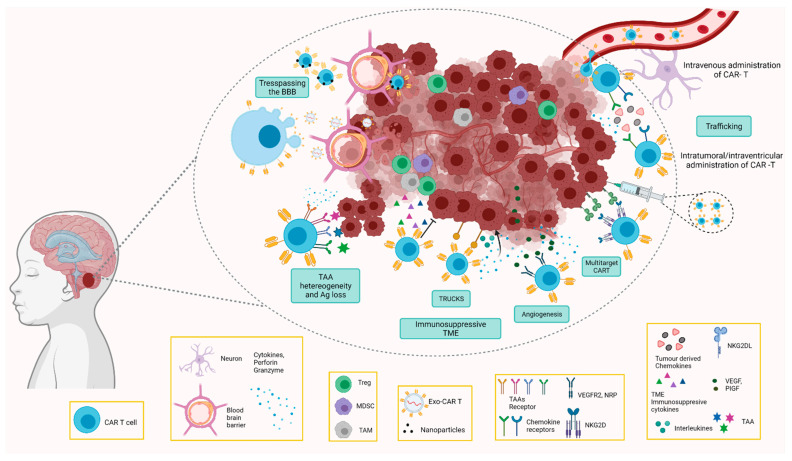
Summary of CAR T cell strategies to improve clinical outcomes in paediatric CNS tumours. Intravenous, intratumoral, or intraventricular delivery routes. Exo-CARs and MNPs carrying the CAR T cells to enhance therapy trespassing the BBB. CAR T cells against immunosuppressive cells and tumour vasculature, inducible release of cytokines by TRUCKS to overcome the immunosuppressive TME. Overcoming the heterogeneity of tumour antigen targeting several antigens. Created with BioRender.com (accessed on 2 October 2021).

The lower clinical success of CAR T cells in solid tumours is most likely multifactorial, but heterogeneous tumour antigen expression, limited infiltration of T cells into the tumour, and the immunosuppressive TME are the main hurdles. In the case of paediatric brain tumours, there are additional challenges, including the anatomical location, the BBB barrier, the specific immune function of the CNS, and the necessity to avoid toxicities. Toxicities may cause long-term sequelae, including not only morbidities and secondary neoplasia, but also social-economic implications [55]. Some of the opportunities presented by CAR T cells are summarised in Figure 1.

#### 4.1.1. Location

Paediatric and adult CNS tumours typically emerge from different tissues. In total, 54 to 70% of all brain tumours in children are present in the posterior fossa (including the brainstem and cerebellum), while only 15–20% of adult brain tumours are in this location [56].

MB, ependymoma, and brain stem glioma are common posterior fossa brain tumours in children. Although these are less frequent in children younger than 1 year of age, the posterior fossa is the most common site of brain tumours in the first decade of life. Tumours occurring in this area are usually of either neuronal or glial origin [57]. Neurosurgery is the mainstay of treatment in posterior fossa tumours in children, with the goal of safe, maximal resection of the tumour. Location is still a challenge for surgical treatment, with the consequent dismal prognosis for unresectable tumours [58].

#### 4.1.2. Blood–Brain Barrier

The BBB is a highly regulated barrier between the brain and other organs to protect the brain from toxins, pathogens, and inflammation. However, simultaneously, it hinders the entry of many treatments. The BBB is composed of capillary endothelial, pericytes, astrocytes, neurons, and the extracellular matrix conforming to a neurovascular unit that protects the brain and maintain homeostasis [59]. Regarding the ability of immune cells to trespass the BBB, the CNS was traditionally considered an immunoprivileged site, but that idea is not accurate anymore [60]. The trafficking of immune cells is tightly regulated [61]. In the absence of neuroinflammation, immune surveillance occurs via draining lymphatics to deep cervical deep nodes, with rare translocation of immune cells across the BBB. These specific immune events occur under precise endothelial cell signalling and immune cell-shape fluctuation, together with an exquisite balance in the expression of adhesion molecules [62,63,64].

In contrast, in response to excessive inflammatory signals, peripheral adaptive and innate immune cells, including monocytes, neutrophils, and B and T cells can enter the CNS, where they execute distinct cell-mediated effects to maintain the homeostasis of the brain and avoid damage from inflammation [60,65]. The CNS-resident immune system is mainly comprised of innate immune cells called resident macrophages and microglia. These myeloid cells are highly specialised but also very plastic, and they respond immediately to any changes in CNS homeostasis, becoming reactive and promoting inflammation [66]. This way, in response to inflammation, including the one produced by tumours’ brain stromal cells, high levels of immunosuppressive cytokines, such as TGFβ or IL-10, are secreted. This secretion favours tumour growth and complicates satisfactory treatment outcomes [60,67,68].

Regarding the BBB permeability, the heterogeneity of paediatric brain tumours must be considered when applying CAR T cell treatments. Phoenix TN et al. showed that, in MB, even within the same group of tumours, the different subtypes differ in the functionality of the BBB presented. Whereas the WNT subtype presents an aberrant vasculature, making a dysfunctional BBB and leading to accumulation of antitumour treatment, the SHH subtype has an intact BBB, making it less susceptible to treatment and, therefore, less curable [69]. Knowing the state of the BBB and how it can be manipulated can enhance CAR T cell therapy treatment.

#### 4.1.3. Tumour Microenvironment

The composition of the TME is crucial to elucidate treatment response to CAR T cell therapies. The TME is formed by a close interaction between tumour and non-tumour cells. Non-tumour cells include microglial cells, endothelial cells, pericytes, fibroblasts, and immune cells. While several of these cell types are also prevalent in brain tumours, some important features distinguish the brain tumour stroma from other tissues. The crosstalk among the different cells and the response to cytokines contribute to tumour growth and outcome to treatment [70]. Different immunosuppressive cells can also be found as regulatory T cells (Tregs), tumour-associated macrophages (TAMs), and myeloid-derived suppressor cells (MDSCs), which express cytokines associated with immune suppression, tolerance, and homeostasis [71]. Additionally, tumour cells can express immune inhibitory ligands that inhibit T cell activity, increase T cell exhaustion and promote the formation of a hostile TME [60]. Microglia are the resident macrophages of the CNS. Under physiological conditions, they exist in a resting state. After a stimulus, they activate, producing proinflammatory cytokines and chemokines to restore brain homeostasis [72]. In response to a microenvironmental signal, macrophages polarise to a different state. This polarisation can be summarised on M1 and M2. Both are key regulators of cancer progression. M1 have antitumor properties, whereas M2 macrophages promote tumour angiogenesis, immunosuppression, and stromal activation. The interaction between tumour cells and macrophages promotes mainly an M2 switch [73].

Comparing with myeloid cells, the presence of T cells in the TME is low, thus contributing to T cell exhaustion and lack of T cell persistence. Besides, the immunosuppressive cytokines released by TAMs lead to T cell senescence [74]

Immune checkpoint inhibitors have emerged as a promising therapy to unblock antitumour T cell responses but have led to some disappointing results in early phase clinical trials for paediatric tumours [4,43,53]. Programmed death ligand 1 (PD-L1) expression in paediatric tumours has been low in general, ranging from 0 to 36% PDL-1 positivity, depending on tumour type.

To properly induce an immune response against a tumour, we need to increase the immunogenicity of the tumour niche. Unlike adult brain tumours, in paediatric patients, the immune cell infiltration has not shown any correlation with tumour grade or mutational load, suggesting new strategies must focus on boosting the antitumour response in the tumour instead of reversing the immune escape mechanisms [7].

Paediatric CNS tumours have characteristics of “cold tumours” phenotype. They present low numbers of TILs with limited activity [2]. Several factors contribute to T cell infiltration, such as cytokines, integrins, tumour neoantigens, and tumour vasculature, having an important role in response to CAR T cell therapy [61].

Recruitment of immune cells to the tumour is also dependent on cytokines and chemokines. The scarcity of these cytokines in immunological “cold” tumours contributes to the maintenance of the immunosuppressive microenvironment and the failure of the immune system to eradicate tumour cells. Approaches that ensure the availability of these cytokines in the microenvironment of tumours without increasing their presence systemically must be developed. Besides, cold tumours lack substantial CD8^+^ T cell infiltration needed for antitumour immune response and surveillance.

Additionally, we need to consider that different paediatric CNS tumours have a distinctive immunophenotype. Pilocytic astrocytoma and ependymoma have higher antitumour T cell infiltration than MB, which is highly immunosuppressed. GBM, ependymoma, and MB exhibit a myeloid immunosuppressive TME, with a high proportion of M2 macrophages and poor recruitment of immune cells. Diao S et al. showed that MBs are cold tumours with a noninflammatory and immunosuppressive TME that leads to poor recruitment of immune cells [75].

TAMs tend to be pro-tumorigenic in brain tumours, producing low levels of proinflammatory cytokines and contributing to low immune T cell infiltration, although their role is still controversial and it seems tumour-context dependent [68]. While, in MB, TAMs have been reported to have an antitumoral role [76], in gliomas, inhibition of TAMs leads to a blockade in tumour progression [76,77]. Furthermore, TAMs contribute to the release of immunosuppressive cytokines, such as IL-10 and TGF-β, that promote tumour growth and decrease response to CAR T cell therapy [68,78].

By contrast, DMGs are not highly immunosuppressive tumours. They include relatively noninflammatory macrophages and low tumour immune infiltration, suggesting limited antitumour immune surveillance [79].

Thus, strategies aiming to reprogramme the tumour immunophenotype and boost immune cell recruitment and activation would increase antitumour immune responses within the TME [68]. Moreover, strategies to increase T cell persistence will enhance CAR T cell therapy.

#### 4.1.4. Lack of Specific Tumour Antigens

The low tumour mutational load in paediatric brain tumours produces few neoantigens, making it more difficult to find targets for CAR T cell therapy, and challenging T cell activation and response to immunotherapy [80]. Accordingly, the low mutational burden will fail in promoting an existing T cell immune response, such as immune checkpoint inhibitors, making it ineffective [2,4]. The lack of specific tumour antigens leads to the development of CAR T cell therapy-targeting TAAs that can also be expressed at low levels on normal cells, leading to toxicities (Table 2). Some clinical and preclinical studies have identified B7 homolog 3 (B7-H3), EGFRvIII, ephrin type-A receptor 2 (EphA2), HER 2, and IL13Ra2 as promising TAAs overexpressed on some paediatric CNS tumours (see Table 2 and references [81,82,83,84,85,86,87]).

CAR T cells targeting HER2, IL13Rα2, EphA2, B7-H3, and disialoganglioside GD2 (GD2) have been performed, showing efficacy [26,27,82,83,84,86,90,91,100,101]. Recently, Haydar D et al. established a hierarchy for the expression of the antigens B7-H3, GD2, IL13Rα2, and HER2, the most common neoantigens expressed on CNS paediatric tumours [102]. The ongoing clinical trials of CAR T cells targeting TAAs in children and young adults with recurrent or refractory CNS tumours are shown in Table 1.

Regardless of the efforts, still antigen escape and lack of CAR T cell persistence are among the main causes of treatment failure [24,103,104,105,106]. In a GMB patient, the intracavitary and intraventricular infusion of IL13Rα2 CAR T cells showed an initial remarkable response for 7.5 months. Although the procedure showed limited adverse effects, the patient eventually relapsed due to antigen-escape phenotype [26]. Additionally, a study in GBM patients treated with EGFRvIII-CAR T cells administered intravenously showed trafficking of the CAR T cells to tumour cells, although the efficacy was inhibited by the local TME and antigen heterogeneity [107].

In the past, the infusion of HER-2 CAR T cells in GBM raised some concerns about efficacy and persistence in some clinical trials. In a phase I clinical trial, anti-HER2 CAR-T cells were administered intravenously in HER2-expressing GBM patients, including some children. CAR T cell persistence was observed for 1 year. About half of the patients showed clinical benefits, tumour heterogeneity being the main obstacle for better response rates [27]. Modifications of the CAR T design and targeting of several TAAs should be devised to maximise efficacy of this therapy [27].

Epigenetic modulators could restore immunogenicity. Paediatric brain tumours carry fewer somatic mutations and more epigenetic alterations than adult ones. The methylation profile represents a preserved molecular memory for the cell of origin and, during the disease, shows a characteristic pattern for each discrete tumour entity. Epigenetic modulators, such as histone deacetylase inhibitors (HDACis) or DNA methyltransferases (DNMTi), in combination with CAR T cell therapies, can increase effectiveness [108].

#### 4.1.5. Toxicity

Although CAR T cell therapies have become a powerful tool in the last decades, with encouraging outcomes, especially in haematological malignancies, they also harbour toxicities that impact the morbidity and occasionally mortality of cancer patients [109,110].

CAR T-cell-associated toxicities are considered unique, in contrast to side effects caused by chemotherapy, which are often nonspecific and can cause permanent multiorgan damage. As a consequence of CAR T specificity, many of its associated toxicities are on-target and occur when CAR T cells are expanding, eradicated, or exhausted [111].

Cytokine release syndrome (CRS) is the most common type of toxicity caused by CAR T cells. CRS can cause headache, nausea, fever, malaise, anorexia, myalgias, hypotension, and can include multiorgan dysfunction and needs urgent intervention [112,113]. The severity of CRS varies among different scales. CRS or related neurotoxicity are other complications that have been observed in patients treated with CAR T CD19 for B-cell acute lymphoid leukaemia [84,114]. Moreover, toxicities related to the on-target off-tumour effect should be considered.

While some studies have correlated toxicity symptoms with in vivo CAR T cell expansion, in others, this correlation is not that clear [115]. The mechanism is not fully understood, but cytokine release by CAR T cells plays a significant role in this syndrome. Among them, IL-6, IFNγ, IL-15, IL-8, IL-10, and IL-2 are found to be elevated in the serum of patients experiencing CRS [112,113,116]. Several reports demonstrated that CRS usually occurs within the first week following CAR T-cell infusion [117,118]. Moreover, Teachey DT and collaborators showed a strong correlation between the severity of CRS and the highest levels of CAR-T cells and serum IL-6 [119]. Furthermore, in leukaemias, studies of Norelli M et al. using xenotolerant murine models and Giavridis T et al. in SCID-beige mice showed that CRS was associated with an increase in IL-1 and IL-6, a hallmark of CRS, and that the monocytes and not CAR T cells were the major source of both cytokines in CRS [120,121].

The second major side effect developed by patients treated with CAR T cell therapies is neurologic toxicity and, in particular, the immune effector-cell-associated neurotoxicity syndrome (ICANS) [110,122]. ICANS is associated with disruption of the BBB and increased cerebrospinal fluid (CSF) cytokine levels, and can present as aphasia, an altered mental state, tremor, seizures, headache, and life-threatening cerebral oedema, often occurring concurrently with or following CRS [123]. The most severe cases of ICANS have been associated mainly with patients who develop CRS and take place at the same time as CRS or some days later. The pathogenesis is even less clear than for CRS but, similarly, ICANS occurs when the peak of CAR T cells is reached [115,124]. Since severe ICANS has been related to increased CSF protein and cytokine levels, it has been suggested that this toxicity is triggered by both an increase in BBB permeability and local production of cytokines by cells within the CNS [91,124].

Other adverse effects regarding the administration of CAR T cell therapies include persistent cytopenias, infections, and tumour lysis syndrome [125,126]. Besides, the on-target off-tumour toxicity is an unavoidable side effect when CAR T cells target tumour-specific antigens. If the tumour-specific antigen is also expressed by tissue stem cells, this toxicity is extremely hazardous, with the risk of tissue destruction [127].

Mount CW et al. reported lethal neurotoxicity after infusion of anti-GD-2 CAR T cells in a patient-derived orthotopic xenograft model with H3K27M+ DMGs of the thalamus. This toxicity was not observed for a patient-derived orthotopic xenograft model of H3K27M+ DMGs of the pons or spinal cord. These findings highlight the importance of tumour location, monitoring, and neurointensive care management [91]. Besides, clinical trials are showing that GD2 CAR T cell therapy for DIPG and spinal cord DMG are safe and show signs of clinical benefit [128]. In some reports, the adverse events caused by the CAR T cells have been related to the tumour burden, suggesting that treatment of small brainstem tumours may reduce CRS or neurotoxicity [91,129]. These results could be life-changing for the outcomes of children with paediatric brain tumours.

### 4.2. Overcoming the Challenges by CAR T Cells

The use of CART cell therapy in paediatric brain tumours can be a useful strategy for those tumours with suboptimal outcomes and treatment possibilities. However, successful clinical outcome strategies that facilitate trespassing the BBB, increase T cell infiltration, and improve in vivo CAR T cell persistence and functionality need to be developed and tailored to the different tumours and subtypes (Table 3 and Figure 1).

#### 4.2.1. CAR T Cells Directed to the Immunosuppressive TME

The immunosuppressive TME hinders the success of CAR T cell therapy. Even in the case that CAR T cells could reach their target antigens, the TME inhibits effector T cell function, hampering their antitumour effect. The brain has immunosuppressive mechanisms to mitigate the inflammatory state [60,67,68]. Poor CAR T cell persistence can inhibit the antitumour effect. Several approaches to increase CAR T cell persistence without increasing toxicities are under investigation. CD28 and 41BB are the main CAR T co-stimulatory domains used. 41BB CAR T cells have shown higher persistence than CD28 costimulatory domain, which is associated with rapid expansion. Other costimulatory domains or the incorporation of both can increase the persistence of CAR T cells in CNS tumours [74].

Moreover, immunosuppressive molecules and cells within the TME can decrease the function and persistence of T cells. TAMs in the brain hamper the availability of cytokines, contributing to low T cell infiltration. CAR T cells targeting TAMs and, therefore, able to reprogramme the TME have been recently developed for ovarian cancer [181]. Moreover, in a recent publication, antiFOLR2 CAR T cells targeting TAM in murine models of ovarian cancer, colon cancer, and melanoma were able to reprogramme the TME and improve the efficacy of CAR T cells [181]. In a murine model of GBM, Li Y et al. designed a TGFβ-resistant EGFRvIII CAR T showing increased survival. They also found polarisation of microglia from a protumorigenic towards a proinflammatory phenotype [138]. The TME can be remodelled by the production of cytokines. Alizadeh D. et al. showed in a GBM model that the increased production of IFNγ by CAR T cells provides immune-stimulatory effects that changed the tumour immune landscape, including both myeloid and lymphoid compartments to promote a more activated and less suppressive TME [182]. Moreover, some studies have shown the role of IFNγ in inhibiting TAM-induced immunosuppression and monocyte differentiation into immunosuppressive M2 macrophage phenotype, thus preventing TAM generation and functions [183].

TRUCKS are CAR T cells engineered with the inducible release of cytokines. CAR T cells releasing IL-12, IL15, and IL-18 have been studied in preclinical models, increasing efficacy even in advanced tumours [130,131].

These cytokines increase IFN-γ secretion, favouring T cell infiltration and persistence, as well as decrease the level of proangiogenic molecules, reactivating the endogenous immune system [132]. For CNS tumours, the local and the controlled administration of these immunomodulatory cytokines is paramount to minimise toxicities, since high toxicity has been related to their systemic application [133].

In a syngeneic model of GBM Agliardi J et al. showed that the local administration of IL12 improved CAR T cell function, eliminating tumour growth. IL12 was also able to modulate the TME, and increase CAR T cell persistence and infiltration of T cells [134]. In a phase 1 clinical trial in patients with HGG, the controlled intracerebral expression of IL-12 was shown to be safe. The production of IL-12 was regulated by a ligand-inducible expression switch and controlled by the oral activator veledimex. The results showed tolerability and an increase in TILs [184]. IL-18 release has also been incorporated in some CAR T therapies. This therapy showed potent antitumour efficacy, both for melanoma [185] and in a pancreatic tumour [130] model of immunocompetent mice. Its functioning has been further explored in other tumour-bearing mouse models, arising as a promising therapy for solid tumours [186]. IL-15, a cytokine with a role in T cell survival and antitumour activity, is an alternative TRUCK strategy. Alizadeh D et al. showed that CAR T cells expanded in IL15 preserved a stem-like memory T cell (T_SCM_) phenotype and improved their metabolic fitness, resulting in superior in vivo antitumour activity [187]. Moreover, GD2 CAR T cells expressing IL-15 were enriched in stem-cell-like cells and promoted enhanced CAR T antitumour effect and expansion in a paediatric tumour model of neuroblastoma [188]. Besides, IL-7 secretion, together with IL-15, has been used in a CAR T strategy to extend T cell persistence [189].

Although many preclinical studies have shown the feasibility of using TRUCKS, further research is warranted in paediatric CNS tumours. The selection of the appropriate interleukin(s) for each tumour type to produce a change in the immune state of the TME is essential to boost CAR T cell effector functions without increasing toxicities [123,133].

Despite several trials that are currently testing armoured CAR Ts, at the moment, none of them are focused on brain tumours [135,136].

CAR T cells designed to express proinflammatory cytokines could balance the microenvironment immune milieu from tolerant to inflammatory, improving antitumour immune response [22]. In this regard, Jin L et al. showed how CAR T cells expressing IL-8 receptors (CXCR1 or CXCR2) increased the intratumoral trafficking of CAR T cells and enhanced antitumour responses in several preclinical models of aggressive tumours, including GBM [137].

Moreover, the platelet-derived growth factor receptor α (PDGFRA) is uniquely expressed by fibroblasts in the adult heart [93,190], and it is dysregulated in a subset of pHGGs and DIPG tumours, driving glioma formation and associated with worse prognosis [46,94,95,96,97]. Xiao W et al. have shown that PDGFRA CAR T cells exhibited potent killing activity toward PDGFRA-positive rhabdomyosarcoma cells in vitro and in vivo; the same approach can be applied to pHGG tumours with activation of PDGFRA [142,191].

Radiotherapy can increase tumour immunogenicity but the neurologic effects and neurocognitive morbidity associated with radio- and chemotherapy are of major concern for the long-term survivors [192].

Gene-editing techniques, such as CRISPR-Cas9, that can disrupt a gene of interest are under investigation. The disruption of T cell inhibitory molecules, such as PD1, CTLA-4, TIM-3, or TIGIT, in CAR T cells can increase persistence and, therefore, treatment efficacy [74]. In this context, in a preclinical model of GBM, Choi BD et al. showed that the intraventricular infusion of an EGFRvIII CAR T resistant to PD1 inhibition prolonged survival and achieved complete and durable cures in some animals [193].

Therefore, CAR T cell strategies should consider these characteristics and address the recruitment, activation, and retention of tumour-specific effector immune cells [79].

Angiogenesis: Abnormal tumour blood vessel is another characteristic of solid tumours, including some paediatric CNS tumours, that hinders CAR T cell infiltration.

Some paediatric brain tumours are highly angiogenic, such as HGG, but the benefit of antiangiogenic therapy in children is not clear. Some clinical trials are studying the efficacy of antiangiogenic treatments on paediatric brain tumours. Although it is a safe procedure, the antitumour effect is poor, and one of the main problems they face is the inability to cross the BBB [194].

As for other approaches, adult GBM has been used as a model for tumour angiogenesis, making the extrapolation of the results to paediatric tumours insufficient [195].

HGGs and grade III MB express high levels of the proangiogenic factor vascular endothelial growth factor (VEGF) and PDGFR [194]. Bao et al. found that glioma cancer stem cells (CSCs) in comparison to non-CSCs, produce elevated amounts of VEGF [196].

Targeting VEGF will not only decrease or normalise the tumour vasculature, but will improve tumour immunity. VEGF functions also as an immunosuppressor molecule that blocks the maturation of dendritic cells, decreases T cell proliferation, and increases the number of Tregs, as well as MDSC activity [139]. Anti-VEGFR2 CAR T will likely improve tumour immunity while normalising tumour vasculature. This treatment seems more indicated to early stages of the disease, although it has not been tested in paediatric CNS tumours yet [140].

Placental growth factor (PlGF) is a member of the VEGF family and is involved in bone-marrow-derived cell activation, endothelial stimulation, pathologic angiogenesis, and wound healing [98,99]. The role of PIGF in tumour promotion is controversial, with some studies showing the involvement of PIGF in tumour growth and others showing the opposite [98]. Around 90% of primary MBs express PIGF, as well as other paediatric brain tumours, and correlate with poor survival [98]. A phase I clinical trial to study different doses of a humanised antibody against PIFG has already been completed in paediatric patients with relapsed or refractory MB (NCT02748135), although no results have been reported. PIGF is also expressed in some other paediatric CNS tumours, such as gliomas, ependymomas, and AT/RT tumours, making this approach a therapeutic opportunity. Targeting the tumour vasculature will decrease hypoxia but also increase oxygen levels, immune cell infiltration, and therapeutic delivery.

This CAR T cell therapy alone or in combination with another strategy, such as immune checkpoint inhibitors, or local CAR T administration could improve survival, as has been shown for other paediatric solid tumours [142].

#### 4.2.2. Trespassing the BBB

Several strategies have been used to facilitate the delivery of therapeutic agents, including transient BBB disruption by using hypertonic solutions or low-intensity pulsed ultrasounds, nanoparticle-based carriers, or the use of alternative routes to CNS drug delivery (intraventricular/intrathecal or olfactory routes) [59,197,198]. In a murine model of glioma, temporal disruption of the BBB by low-intensity pulsed ultrasound increased the delivery of EGFR CAR T cells and prolonged survival of the treated mice [199]. Other approaches have emerged with real possibilities in paediatric CNS tumours.

##### CAR T Cell Delivery

With an inefficient effector cell trafficking, only a minor fraction of CAR T cells can be found infiltrating the tumour [154]. Nanoparticles are one of the innovative approaches that can be used as carriers for drug delivery [154]. Their physical and chemical characteristics and the possibility to attach different molecules make them an interesting tool to transport the therapeutic compound across the BBB [155]. Metallic, polymeric, and lipid nanoparticles can be used to transport the therapeutic compounds, stimulate CAR T cells before infusion, or overcome TME immunosuppression. Moreover, their pharmacokinetic and pharmacodynamic properties allow the use of low doses of immune-modulating molecules, reducing their side effects [154].

Very few studies have been explored with the use of nanoparticles for enhanced CAR T cell therapy in brain tumours, most of which have been summarised by Balakrishnan PB et al. [155].

Recently, one study revealed that a nanoparticle RNA vaccine can directly enhance the cytotoxic effect of CAR T cells and overcome insufficient stimulation and low sustainability [156].

A different approach has arisen in the last years for the use of nanoparticles. Loading CAR T cells with magnetic nanoparticles (MNPs) can increase the delivery to the tumour site, therefore, overcoming the anatomical barriers. The adsorption of effector cells to MNPs to guide and retain them using an external magnetic field (EMF) at the tumour site could increase CAR T cell infiltration and decrease CRS toxicity by reducing the number of effector cells infused [157]. This fact is very important in children, since sometimes it is difficult to obtain enough material for the generation of autologous CAR T cells for brain tumours. Sanz-Ortega L et al. showed that MNPS attached to the T cell surface can be guided and retained to the target site by an EMF without affecting their biological activity and characteristics [157]. They also showed that CD8^+^ T cells loaded with MNPs can be directed to a tumour expressing an antigen of interest [200].

MNPs technology has already been approved for the treatment of brain tumours by inducing intratumoral thermotherapy in patients with recurrent GBM multiforme [201].

Magnetic hyperthermia takes advantage of the susceptibility of cancer cells to high temperatures. This way, intratumorally injected MNPs generate heat after exposure to an external alternating magnetic field and, consequently, induce cell death within tumours. An advantage of this technique is the application to unresectable or difficult to access brain tumours. Another important point of the use of MNPs is that they can restore cancer-specific immune responses [202].

Although this approach can solve some of the main limitations of CAR T cell therapy, much work should still be done to understand the mechanisms of interaction between T cells and MNPs, and optimise delivery.

##### CAR T Cell-Derived Exosomes

Exosomes are small vesicles (30–120 nm) containing nucleic acid (DNA, mRNAs, and microRNAs) and protein cargo secreted by all cell types. They are found in body fluids, including blood, saliva, urine, breast milk, and CSF. Exosomes are viewed as specifically secreted vesicles enabling intercellular communication [145].

Recent publications have shown that exosomes derived from CAR T cells (Exo-CARs) hold great potential to target cancer cells [146,147,148]. Exo-CARs maintain the same membrane topology as CAR T cells, with the extracellular domain exposed on the surface of the exosomes, and, thus, keeping the antigen recognition capacity. Additionally, upon antigen recognition on the tumour cell, Exo-CAR releases cytotoxic effector molecules, such as granzyme B and perforin, and exerts antitumour cytotoxicity. Besides having the same antigen recognition and antitumour toxicity of CAR T cells, they possess some advantages over their parental cells. Their nanoscale size would confer an improved ability to cross this barrier over the whole cell [149,203,204]. Additionally, Exo-CAR may easily penetrate the stroma-rich matrixes and may be more helpful to treat solid tumours [147,148]. Moreover, recent studies by Fu W et al. and Yang P et al. in murine models of solid tumours showed that animals injected with Exo-CAR exhibited no signs of toxicity, even at the highest dose tested [147,148]. Furthermore, one of the studies showed Exo-CARs lack expression of inhibitory molecules, such as PD1, protecting them from T cell exhaustion and providing enhanced resistance to the immunosuppressive TME [148]. Recently, Exo-CD19 CARs have been derived from CD19CAR-expressing HEK293T cells. This strategy aims to develop “off-the-shelf” targeted CAR exosomes, avoiding the limitations of manufacturing CAR T cells from autologous PBMCs [146]. It is important to note that both platforms—CAR-T-derived exosomes and CAR T cells—can be combined and/or alternated, and this combination will probably strengthen the application for CAR-based cancer therapy in paediatric CNS tumours. However, the use of exosomes equipped with CAR molecules is still at a starting point and needs further exploration.

##### Routes of Administration of CAR T Cell Therapy

Delivery of CAR T cells in paediatric brain tumours is also a topic of debate. Different routes have been studied: intravenous, intraventricular, and intra-tumour-cavity, with the results favouring the last two routes. In a preclinical model of AT/RT tumours, the intraventricular or intratumoral administration of B7-H3 CAR T cells had a higher antitumour effect and reduced systemic levels of inflammatory cytokines when compared to CAR T cells administered intravenously [150]. In a xenograft mouse model of MB and ependymoma, the administration of EPHA2, HER2, or ILRα13 CAR T cells in the CSF was an effective treatment, increasing the amount of CAR T cells in contact with the tumour cells and decreasing toxicities [85].

For these two types of tumours that metastasize adjacent to the CSF, this could be the preferred administration route to decrease toxicity and bypass the BBB.

Systemic delivery of CAR T cell therapy for CNS tumours could lead to inefficient crossing of the BBB. Mulazzani et al. showed that the intravenous administration of CAR T cells was less efficient in tumour killing than the intratumor administration [151]. Nellan et al., in a xenograft murine model using different MB cells lines, showed that the HER2-BBz-CAR T cells effectively eliminated tumour cells via regional and intravenous delivery, although intravenous delivery required a high concentration of effector cells [152]. Since a high concentration of effector cells could increase on-target off-tumour toxicities, locoregional delivery is the preferred route of administration in most clinical trials (Table 1). Moreover, other authors, such as Priceman PJ et al., demonstrated that intraventricular regional administration of HER2-CAR T cells was more effective and with fewer side effects than local delivery, using an orthotopic human xenograft model of breast cancer metastasis to the brain [153]. Limited investigations are available for brain tumours but, reiteratively, the local intracranial CAR T administration seems to be a better option than intravenous infusion, and, also, regional delivery could be beneficial over local infusion [205].

## 5. Antigen Escape

Molecular and cellular heterogeneity is one characteristic of brain tumours that hampers CAR T cell therapy. Even in the case of a uniformly expressed TAA, there is the possibility of antigen loss. Antigen escape is one of the major challenges in CAR T cell therapy, with tumour cells selecting clones that downregulate antigens targeted by effector CAR T cells.

The ideal target should be expressed on most tumour cells and, specifically, in the cancer-initiating cells, which are usually resistant to conventional therapies [206,207,208,209].

c-Met, CD133, and CD171 are CSC markers expressed on GBM. In GBM, CAR T cells targeting CSC markers have had remarkable success in preclinical studies [164]. Although some clinical studies are ongoing, none of them are being developed in brain tumours [165].

The tumour antigens should not be expressed on cells from healthy tissue, avoiding toxicity through on-target off-tumour effect. However, even when the target has been identified and it is mostly expressed, malignant cells can outgrow at relapse, downregulating TAA expression, which, in the case of CD19 CAR T for haematological malignancies, has been associated with splicing alternatives [158,210].

Moreover, CAR T cells require higher antigen densities to fully activate effector functions [159,160,161,211]. The heterogeneous expression of target antigens in the tumour, along with the immunosuppressive TME, lead to tumour CAR T cell killing escape when using a single antigen. One strategy to overcome this limitation is the design of CAR T constructs targeting several antigens. NKG2D CAR T cells recognise up to six ligands (NKG2DL) that are usually overexpressed on tumour cells and cells from the TME but not in healthy tissue [88,162]. We have previously shown that these ligands are upregulated in MB [89]. Moreover, we proved the safety and effectiveness of NKG2D CAR T cells in an in vivo model of paediatric osteosarcoma [163]. Although NKG2DL shedding has been studied as an immune escape strategy in some tumours, these ligands were not detected in serum when a cohort of paediatric brain tumours was analysed [7]. Specifically, NK cells have been shown to efficiently target paediatric DIPG tumours, reinforcing the anti-tumour effectiveness of NKG2D CAR T therapy [79].

Advances in RNA sequencing, microarray analysis, and proteomics have made it possible to identify new preclinical targets. This way, strategies that combine different approaches or CAR T cells targeting multiple antigens should be considered.

Other alternatives are to study the antitumour effect of CAR T cells expressing membrane-anchored gangliosides. One of the most studied in paediatric tumours is GD-2. GD-2 is expressed at low levels on some tissues [92,101] (Table 2). GD-2 CAR T is already in clinical trials in neuroblastoma, osteosarcoma, and H3K27M+ DMGs [28,127,212] GD-3 is another ganglioside highly expressed on malignant gliomas; thus, this could be another candidate for CAR T cell therapy in children with CNS tumours [166].

As we previously mentioned the possibility of targeting TAMs expressing folate receptor β by an anti FOLR2 CAR T, some tumours are also positive for folate receptor α (FRα). In 95 MB patients, Liu et al. observed expression of FRα, comparing with healthy brain tissue; also, in an in vivo model, tumour growth decreased upon FRα targeting [143,144].

## 6. Toxicity

For CNS tumours, CAR T cell therapy-related toxicity could lead to catastrophic outcomes and long-life side effects, so minimisation of adverse events is critical. Preclinical research is providing novel alternatives to reduce CAR-T-associated toxicities that have to be cleared in clinical trials. The intensity of conditioning therapy, high CAR T cell dose, and CAR T cell construct design are among the main factors that lead to increased CAR T cell expansion in vivo and boost toxicity.

As an example, some groups are focusing their research on modifying the CAR structure to ameliorate toxicity by decreasing CAR-antigen binding domain affinity to micromolar affinity or altering CAR transmembrane regions to modulate cytokine secretion [213]. Other methods involve the modification of CAR T cells expressing suicide switch molecules, CAR T cells directed against tumour antigens, or pharmacological immunosuppression by using immunomodulatory pharmacologic drugs [172,173,174,175,176]. Several different approaches are already under investigation, with some of the systems being in early phase clinical trials. For paediatric CNS tumour patients, several considerations must be considered when choosing the approach. In case of life-threatening toxicity, iCasp9 irreversibly eliminate CAR T cells. Endogenous switches, such as synNotch and iCAR, regulate CAR T cells, but the time and intensity of CAR T cell activity cannot be controlled [177]. Some strategies have already been tested in clinical trials in CNS tumours, such as the CAR T expressing the gene coding for inducible caspase-9 (iCasp9). Phase I efficacy and safety trials of the CD2 CAR T iCasp9 technology have been initiated in several indications, including DIPG and spinal DMG [172] (Table 1).

New methods to keep a balance between cytokine secretions and CAR T cell activation without reaching a toxicity level are needed.

Preclinical models have their limitations. Patient-derived xenografts (PDXs) have been increasingly used in translational research, but immunocompromised models create a host environment that does not recapitulate the one from the patients. The lack of an immune system hampers the potential development of some of the most common negative effects of CAR T cell therapy, on-target off-tumour effect, CRS, and neurotoxicity [214]. Adequate preclinical models should be chosen to better study brain malignancies [215,216,217,218]. Humanised mouse models are probably a better model to recapitulate T-cell-based therapy, but they also present some limitations, such as lack of recapitulation of CAR T cell toxicities; also, these models are expensive and difficult to obtain for routine use [219]. Syngeneic models are more economic and have a functional immune system. They also can unveil on-target off-tumour toxicities. These models present some disadvantages due to the differences in mouse and human biology, including the absence of human antigen targets and different inflammatory environment [220,221]. Moreover, chemokines involved in infiltration can be species-specific, limiting efficient trafficking, i.e., human IFNγ secreted for T cells, a cytokine important for tumour elimination, does not act on murine tumour stromal cells [222]. All these issues do not support the results for clinical translation.

Mathematical modelling has emerged as a tool to predict treatment response and CAR T cell dynamics. This tool can give answers about CAR T cell dosing to avoid relapse and toxicities, studying CAR T cell expansion and exhaustion and the interaction between CAR T cells and immune cells. These aspects are patient-dependent and an important feature of the success of this therapy [178]. In some studies, developed in paediatric leukaemia, the authors underlined the importance of the characteristics of the infused CAR T cells. Leon-Triana O et al., in a pilot mathematical model, used a dual target for tumour cells and the antigen present in normal cells, such as CD19^+^. They showed poor CAR T cell persistence due to the immunosuppression and low levels of tumour target [179]. In brain tumours, mathematical modelling has also been applied in a model of GBM. Sahoo P et al. showed, in a CAR T cell treatment response in glioma (CARRGO) model, that the rate of cancer cell killing by CAR T cells is inversely related to the CAR T cell dose, but CAR T cell dose correlates with the proliferation and exhaustion of CAR T cells [180]. Although with some limitations, such as tumour heterogeneity and immunosuppressive TME, these in silico models are useful for predicting the behaviour of CAR T cell therapy and tumour growth.

## 7. Other Considerations

Optimisation of the CAR T cell manufacturing process to enrich for the population, which can contribute to sustaining an effective antitumour response, is crucial, especially when they encounter hostile conditions of solid tumours [167,182]. To improve the protocols for culturing time, cell density, choice of IL, including concentration and timing of supplementation, for ex vivo culture are important variables to produce an optimal clinical product [103]. Moreover, the selection of a T cell subtype with a non-alloreactive phenotype, such as memory T cells, will contribute to decreasing toxicities. Most clinical trials have been performed with CAR T cells with an effector memory phenotype, which translates to poor persistence in vivo. This lack of persistence has encouraged finding a subset of T cells that are less differentiated or with a memory phenotype [168]. T_SCM_ are under investigation given their self-renewal capacity, engraftment potential, and ability to generate other T cell subsets. Because the frequency of these T cell subpopulations in peripheral blood is very low (2–3%), specific manufacturing processes that can enrich and expand CAR T_SCM_ cells are already in preclinical studies [167,169,170]. Another important question is the therapeutic window of CAR T cell therapy. The density and expression on healthy tissues of TAA for CAR T cells have to be considered. The level of TAA expression on healthy tissues narrows the therapeutic window of this therapy. Moreover, the TAA density threshold for CAR T cell recognition and activation are important issues to be considered. These and other modifications that can broaden this window would be necessary to enhance efficacy [171].

## 8. Conclusions

Little improvement has been made in the last decades for recurrent or relapsed paediatric brain tumours. Children with CNS tumours present unique challenges due to their brain development and immature immune system. The research field in CAR T cell therapy has advanced extraordinarily, but mainly for adult patients and haematological malignancies. Given the differences in paediatric and adult CNS tumours, the results obtained from adults cannot be applied to children. Therefore, new strategies must be tailored to the unique characteristics of paediatric CNS tumours.

Future strategies with CAR T cells need to consider the hurdles of these tumours, lack of specific TAA, tumour heterogeneity, diffusion through the BBB, the TME, and the small number of patients that limit data generation. Innovative therapies, such as Exo-CAR T and nanotechnology, are promising fields due to their characteristics and advantages.

The integration of multidisciplinary data and preclinical research with the combination of different strategies will be required to maximize positive results. All these efforts will translate into breakthroughs in CAR T cell therapy for these patients with generally suboptimal clinical outcomes. Safety should always be the main consideration when assessing new treatment for these children.

## Figures and Tables

**Table 1:** Recruiting or active clinical trials using CAR T cells in children with CNS tumours.

Age	NCT	Phase	Tumour Type	Target Antigen	Administration	Sponsor
2–30 years old	NCT04196413	1	DIPG and DMG	GD2	Intravenously	Lucile Packard Children’s Hospital
1–26 years old	NCT04185038	1	DIPG/DMG, and recurrent or refractory Paediatric CNS tumours	B7-H3	Catheter into the tumour or ventricular system	Seattle Children’s Hospital
1–18 years old	NCT04099797	1	DIPG, Embryonal Tumour, HGG, medulloblastoma	GD2	Intravenously	Texas Children’s Hospital
1–26 years old	NCT03638167	1	Recurrent or refractory Paediatric CNS tumours	EGFR	Tumour resection cavity or the ventricular system	Seattle Children’s Hospital
1–26 years old	NCT03500991	1	Recurrent or refractory Paediatric CNS tumours	HER-2	Tumour resection cavity or into their ventricular system	Seattle Children’s Hospital
4–70 years old	NCT03638206	1 and 2	Several solid tumours. Gliomas from the CNS	EGFR V III	Intravenously	Shenzhen BinDeBio Ltd.
12–75 years old	NCT02208362	1	Recurrent or refractory malignant glioma	IL13Rα2	Intracavitary, intratumoral or intraventricular	City of Hope Comprehensive Cancer Center
4–25 years old	NCT04510051	1	Recurrent or refractory brain tumours in children	IL13Rα2	Intraventricular	City of Hope Medical Center National Cancer Institute (NCI)
1–22 years old	NCT04903080 *	1	Ependymoma	HER-2	Intravenously	Pediatric Brain Tumor Consortium Texas Children’s Cancer Center Baylor College of Medicine
3 years old and older	NCT02442297	1	CNS tumours	HER-2	Tumour, tumour resection cavity, and/or cerebrospinal fluid (CSF) space	Baylor College of Medicine

* Not yet recruiting.

**Table 2:** Most common tumour antigens for CNS paediatric brain tumours.

Target	CNS Paediatric Tumour Expression	Expression in Healthy Tissues	Bibliography
B7-H3	Moderate-to-high levels in MB, ependymoma, and gliomas	Very low or undetected	[81,82,83]
HER2	40% of MB	Not expressed in healthy brain tissue	[84]
IL-13Ralpha2	Highly expressed (60–100%) in gliomas, MB, and ependymoma	Not expressed in healthy brain tissue or other organs except testis	[85,86,87]
EphA2,	Gliomas	Not expressed in healthy brain tissue	[85,86]
NKG2DL	Variable expression in MB	Rarely detectable in healthy tissue	[88,89]
EGFRvIII	Variable expression in pHGG	Not expressed in healthy tissue	[90]
GD2	High levels in pHGG	GD2 in normal tissues is limited essentially at low levels on neurons and peripheral nerve fibres, dermal melanocytes, lymphocytes, and mesenchymal stem cell	[91,92]
PDGFRA	Variable expression in pHGG	In healthy tissues expressed on development	[93,94,95,96,97]
PIGF	Highly expressed in MB	Expressed in placenta and at low levels in several other organs, including the heart, lung, thyroid, skeletal muscle, and adipose tissue under normal physiological conditions	[98,99]

**Table 3:** Challenges for CAR T cell therapy in paediatric CNS tumours and overcoming strategies.

Challenges	Potential Strategies
Tumour microenvironment	Modulation of the TME using TRUCKs [130,131,132,133,134,135,136] Chemokines [137] Blocking immunosuppressive molecules: TGFβ-resistant [138] VEGFR2 CAR T [139,140,141] PDGFRA CAR T [94,95,96,97,142] CAR T cells targeting TAMs [68,76,77,143,144] PIGF CAR T [98]
Trespassing the BBB	CAR T derived exosomes [145,146,147,148] Local delivery [149,150,151,152,153] Nanoparticles [154,155,156,157]
Antigen escape	Targeting multiple antigens [88,89,158,159,160,161,162,163] synNotch CAR T [29] Targeting the CSC [164,165] New neoantigens [166]
CAR T associated toxicities	T cell subpopulation [167,168,169,170,171] iCas9 CAR T [172,173,174,175,176,177] Prediction with mathematical modelling [178,179,180] Manufacturing process [102,168]
